# The Hospital Reform Association

**Published:** 1897-10-30

**Authors:** 


### THE HOSPITAL REFORM ASSOCIATION.

A conference convened by the Hospital Reform Association to consider the question of the administration of medical relief in the out-patient and casualty departments of hospitals was held at St. Martin's Town Hall on Thursday, the 21st inst., the Earl of Stamford presiding. The attendance, especially at the evening meeting, was not large. Amongst those present were: Sir W. H. Broadbent, Bart., Sir Henry Burdett, K.C.B., Mr. Timothy Holmes, Mr. R. Ward Cousins (Chairman of the Hospital Reform Association), Mr. T. Garrett Horder (Hon. Secretary of the Association), Mr. Victor Horsley, F.R.S., Lieut.-Colonel E. Montefiore, and Mr. C. S. Loch (Charity Organisation Society), Drs. Glover, Heron, Herringham, Beverley (Norwich), Herbert Snow, Snape, and Lovell Drage, Mr. Douglas Dent, Mr. Conrad Thies (Royal Free Hospital), Rev. Nathaniel Bromley (King's College Hospital), Mr. Adrian Hope (Great Ormond Street Children's Hospital), and Mr. Henry N. Custance (Hospital Sunday Fund).

The Chairman supposed that few would deny that some reform in hospital administration, and more especially in the out-patient and casualty departments, was needful. From statistics supplied by Mr. Horder it appeared that in London quite a million out-patients were treated at the voluntary hospitals and non-provident dispensaries in the year 1895, and it was scarcely possible for him to believe that all of this million were proper subjects for free medical relief. An inquiry undertaken by the Charity Organisation Society a few years ago had elicited that only one quarter of the people treated in the out-patient departments were appropriate to that treatment. The remainder ought to have gone either to the general practitioner, or to the provident dispensaries, or to the poor law.

Mr. T. Garrett Horder said that the remedies for the present state of affairs must include (1) the abolition of payments by patients and subscribers' letters; (2) the making the out-patient rooms of a more consultative nature than they are at the present time, which could be brought about by the closer union of the hospitals with the provident dispensaries; (3) the foundation of a series of provident dispensaries; and (4) the establishment of a hospital board in every town.

Mr. Timothy Holmes said that nothing more could be added even in the present day to what had been so ably set out thirty years ago by Sir William Fergusson's committee, of which he was a member: the abuses which existed then existed now. The only change was that the abuses had grown and a new form of abuse had sprung up in the shape of enormous casualty departments. At the present time, at most hospitals everybody who applied was seen; and, although he did not attach much importance to the stories that were told of ladies who came to the out-patient departments in their carriages, yet in many ways the out-patient departments were greatly abused. Those who wished to initiate reforms, however, met with an amount of resistance which he believed would be almost insuperable unless their efforts were backed up by the general support of the medical profession. At the present time opinions were so immensely divergent that, although he believed everyone was anxious to do his best, it would be found rather difficult at first to get any movement at all, and when they did begin to move they must be content to move slowly. He proposed the following resolution: "That this conference is of opinion that reform is urgently needed in the methods of administering out-patient and casualty relief in the hospitals and dispensaries of the United Kingdom, and that it be referred to the Council of the Hospital Reform Association to form a committee to draw up a scheme for the better administration of such relief for the consideration of a future meeting of the conference."

Dr. Glover, in seconding, said that the abuses of the out-patient department were growing year by year. If they could get the consulting staffs of the hospitals to see the evils of the present system, and to see that these evils were maintained by their reputation, by their gratuitous services, and by their exhausting hours of unnecessary labour, he believed some satisfactory solution to the question might be found.

Sir W. H. Broadbent, Bart., believed that from several different points they were approaching, in the near future, he would not say a solution of the out-patient problem, but at any rate a careful consideration of it, which in the long run would bring about the results which they all desired to see, namely, the limitation of hospital patients to those who really needed relief, *i.e.*, those who lay between the pauper and the person able to pay a medical man.

Sir Henry Burdett said that this proposal for yet another committee to make more inquiries, to go over old ground, to take up old facts, and to report on the lines which had been gone over before, was not the way to solve the out-patient question at all. They would never get a better or a more impartial investigation than that carried out by Sir William Fergusson's Committee in 1870. Those who read that report, and the documents which accompanied it, and who, at the same time, had a knowledge of the present-day working of hospitals, and the difficulties and conflicting points which attached to the question of out-patient relief, must agree that in that classic document there was every material fact which every other committee could bring out. Therefore, if they appointed a committee, it should not be a committee to hold another investigation, but one which would stand on the old facts, which no one disputed, and would bring together those people and bodies which had it in their power to bring about a new system in the out-patient department. He had come to the conclusion that some system must be planned which would enable the hospitals to say, "We will only give relief to those people who are unable to pay for medical attendance, unless the nature of the case is such that they require such medical attendance as they can only get in a hospital, and if they want such attendance (their social position being above the level of those who ought to have such relief) they must show that they have continuously and regularly given, according to their means, to the hospitals." Of course, that was only another way of applying the provident principle to hospitals, and from his experience of the attendants at out-patient departments he had no doubt that, with the exception of about 10 per cent., those who attended these departments could afford if they chose to pay something for the medical treatment which they received. This suggestion of his could be worked out by means of the Prince of Wales's Hospital Fund subscription books. He would suggest the adjournment of the conference to the beginning of 1898 so that they might see what would be the outcome of this the Diamond Jubilee year.

Mr. R. Ward Cousins thought that the abuses from which hospitals suffered recoiled on the heads not only of the medical profession, but also of the charitable public.

Dr. Beverley (Norwich) said that in the provinces just the same difficulties had to be faced as in London. At the Norfolk and Norwich Hospital they had a committee before which all applicants had to appear, but the name of the nominee whose signature appeared on the ticket carried far more weight with the committee than the individual who bore it.

Dr. Glover pointed out to Sir Henry Burdett that the real object of the resolution was not to appoint a committee to make inquiries, but a committee to devise a remedy.

Dr. Herringham said that, as far as his experience went, there was not so much abuse as people endeavoured to make out. During a few weeks he had carefully watched the cases in the casualty department at St. Bartholomew's, and out of the five or six hundred cases which had passed through his hands only about five or six had been cases which, in his opinion, abused the hospital. So that he thought that, in a properly conducted hospital with an efficient inquiry officer, the amount of abuse could be reduced to the very lowest possible limit.

Mr. Douglas Dent thought that in a great question like this they ought not to introduce too many details. Let them put the principle distinctly before the public, get that accepted, and afterwards thrash out the details.

The Chairman put the proposition to the meeting, and it was carried, about 12 voting for and 6 against it.

The conference then adjourned.

On the conference reassembling,

Mr. Conrad Thies referred to a conference of hospital managers which had gone into this matter of the out-patients recently, and which had made certain suggestions which practically covered the points raised. If the Hospital Reform Association could bring the medical men—consultants as well as general practitioners—to some common understanding on the matter, he was sure that the lay portion of the managers of hospitals would do their best to fall in with the wishes expressed to them by the medical staff. At the present time the greater number of medical men were not in accord on even the question of limiting the number of out-patients.

The discussion was carried on by Drs. Herbert Snow, Snape, Lovell Drage, Woodley Stocker, and Mr. C. S. Loch.

Mr. Victor Horsley re-moved the resolution which is given above. He said there was such a divergency of opinion at the present time that it would, he thought, be better to take this subject piecemeal. In the previous discussion too much stress had been laid on the interests of the medical man and the interests of the public, and too much of an attempt had been made to play one class off against the other. As a matter of fact, the interests of the two classes were coincident. He did not believe that a matter of so much difficulty was to be settled by means of one agency. There must be a number of lines of treatment which must be made to converge upon the evil.

Dr. Heron, who seconded, was sure that if the facts were laid before the good men of the profession it would very soon be apparent to them that it was a desirable thing, and, in fact, their duty to assist in having this matter remedied speedily. He thought that it was the Hospital Sunday Fund and the Prince of Wales's Fund, having the power of the purse, which would prove to be the strongest of the items which would eventually solve the out-patient problem.

Mr. Adrian Hope denied that at the London hospitals there was a gross abuse of charity.

Sir Henry Burdett urged the conference to alter their resolution. Most of the speakers had admitted that the problem was one which must be taken in portions. The Hospital Reform Association was an association composed mainly of medical men, and they might do an immensity of good and help forward the work of remedying the present evils by devoting the work of their committee to communicating by deputations of medical men with the honorary medical staff of the principal London hospitals on the lines he had explained in the *Lancet* a year ago. In this way the Association would be converting the medical profession, whilst at the same time the matter was being considered by the managing committees of the hospitals.

Mr. Victor Horsley did not think, as that resolution had been carried in its present form at the previous sitting, that it could be altered now.

The Chairman agreed with Mr. Horsley, but was sure that the members of the Association would take note of what Sir Henry Burdett had urged.

The resolution was then put and carried, 10 voting for and none against it.

On the motion of Mr. Horder, seconded by Mr. Thies, a vote of thanks was given to the chairman, and the conference broke up.

